# Zinc Chloride Supplementation During In Vitro Fertilization Reduces Polyspermic Fertilization of Porcine Oocytes

**DOI:** 10.1111/asj.70095

**Published:** 2025-08-27

**Authors:** Supitcha Kaewma, Takeshige Otoi, Oky Setyo Widodo, Megumi Nagahara, Aya Nakai, Suong T. Nguyen, Yuichiro Nakayama, Theerawat Tharasanit, Maki Hirata, Fuminori Tanihara

**Affiliations:** ^1^ Faculty of Veterinary Science Chulalongkorn University Bangkok Thailand; ^2^ Faculty of Veterinary Science Prince of Songkla University Hat Yai Thailand; ^3^ Bio‐Innovation Research Center Tokushima University Tokushima Japan; ^4^ Faculty of Veterinary Medicine Universitas Airlangga Surabaya Indonesia; ^5^ Joint Graduate School of Veterinary Sciences Yamaguchi University Yamaguchi Japan

**Keywords:** embryo development, in vitro fertilization, monospermy, pig, zinc

## Abstract

As zinc is important for the fertilization competency of sperms and oocytes, we investigated the effects of zinc chloride (ZnCl_2_) supplementation during in vitro fertilization (IVF) on porcine oocyte fertilization and development. We evaluated the effects of ZnCl_2_ concentration (0, 1, 10, and 20 μg/mL) on the quality of frozen‐thawed boar spermatozoa cultured for 5 h and on oocyte fertilization and embryo development after IVF. Spermatozoa from three different boars were additionally tested. ZnCl_2_ supplementation effects along with metal chelators, calcium ethylenediaminetetraacetic acid (Ca‐EDTA) and zinc EDTA (Zn‐EDTA), were also examined. ZnCl_2_ supplementation did not affect the quality of frozen‐thawed spermatozoa after culture for 5 h. Supplementation with 1‐μg/mL ZnCl_2_ decreased the percentage of polyspermic fertilization compared to that at 0‐ and 20‐μg/mL ZnCl_2_. Moreover, it increased blastocyst formation rate compared to that in the other supplementation groups. In different boar spermatozoa, ZnCl_2_ supplementation (1 μg/mL) decreased polyspermic fertilization but did not improve embryo development. Co‐incubation with Ca‐EDTA did not reduce polyspermic fertilization, but Zn‐EDTA co‐incubation reduced polyspermic fertilization similar to that with ZnCl_2_ alone. In conclusion, 1‐μg/mL ZnCl_2_ supplementation during IVF reduces polyspermic fertilization but may not improve embryo development.

## 1 Introduction

In vitro porcine embryo production is a highly advantageous assisted reproductive technology (ART) offering numerous benefits in various fields, including animal husbandry and human health. In vitro fertilization (IVF) has recently become an integral component of biomedical research because it enables the production of genetically modified pigs that can serve as tissue sources for xenotransplantation and as research models for human genetic diseases. However, polyspermy remains a significant obstacle for achieving optimal efficiency in porcine IVF and in vitro production (IVP) systems (Dang‐Nguyen et al. 2011; Himaki et al. 2025). Therefore, the development of strategies to prevent polyspermy is crucial to overcoming this challenge and maximizing the potential of reproductive biotechnology.

Zinc is an essential trace mineral that plays a significant role in both male and female reproduction by supporting cellular development, gene expression, and RNA polymerase activity (Allouche‐Fitoussi and Breitbart 2020; Garner et al. 2021). In male mammals, high levels of zinc are usually found in the spermatozoa and seminal fluid, though zinc concentrations in the semen vary among species (Allouche‐Fitoussi and Breitbart 2020). Additionally, zinc ions play a vital role in sperm capacitation, regulating key events responsible for fertilization competency (Kerns et al. 2018; Zigo et al. 2022). In female mammals, zinc is involved in follicular growth, germ cell development, meiosis arrest, oocyte maturation, fertilization, oocyte activation, and polyspermy block (Garner et al. 2021). After sperm‐oocyte fusion, calcium oscillations occur to induce a zinc spark, i.e., the rapid release of zinc to the extracellular environment (Que et al. 2017). This zinc spark event is crucial for establishing the zona pellucida (ZP) block, which reduces the number of sperm penetrations through the ZP to reach the fertilized oocyte (Que et al. 2017). To date, zinc supplementation in embryo production has been studied in various species. In cows, adding 1.1‐ and 1.5‐μg/mL zinc during in vitro maturation (IVM) improved preimplantation embryo development, including the blastocyst formation rate and blastocyst cell number (Picco et al. 2010). In yaks, 1‐ and 2‐μg/mL zinc supplementation during IVM increased the blastocyst formation rate and reduced reactive oxygen species (ROS) levels by increasing the amounts of glutathione and superoxide dismutase (Xiong et al. 2018). In pigs, supplementation with 0.8‐μg/mL zinc during in vitro embryo culture improved blastocyst formation (Jeon et al. 2015). Overall, these studies suggest that adding zinc during IVF could improve both fertilization outcomes and embryo growth. However, these potential effects have not been explored in porcine IVF procedures.

In this study, we examined whether supplementation with zinc chloride (ZnCl_2_) during IVF affects porcine oocyte fertilization and embryo development. Further, we validated the impact of ZnCl_2_ supplementation during IVF by employing ethylenediaminetetraacetic acid (EDTA), a metal‐binding agent.

## 2 Materials and Methods

### 2.1 General

Unless stated otherwise, all chemicals used in this study were purchased from Sigma‐Aldrich (St. Louis, MO, USA).

### 2.2 Semen Collection and Cryopreservation

Semen from four Large White boars (A–D) was collected and cryopreserved as described previously (Namula et al. 2018) with minor modifications. Briefly, samples were diluted threefold with Modena extender, transported within 2 h, and centrifuged at 550 ×*g* for 10 min. After removing the supernatant, pellets were resuspended in the first extender at 25°C to a concentration of 4 × 10^8^ cells/mL. The first extender included 0.4‐mg/mL d‐fructose (Fujifilm Wako Pure Chemical Corporation, Osaka, Japan), 2.9‐mg/mL Tris(hydroxymethyl)aminomethane (Fujifilm Wako), 1.59‐mg/mL citric acid monohydrate (Fujifilm Wako), 0.2‐mg/mL amikacin sulfate (Meiji Seika Pharma Co. Ltd., Tokyo, Japan), and 20% (v/v) egg yolk. The samples were then cooled for 2.5 h at 5°C, followed by adding the second extender (the first extender supplemented with 6% [v/v] glycerol [Fujifilm Wako] and 1.48% [v/v] Equex STM Paste, Minitube USA, WI, USA). Spermatozoa were frozen in 0.25‐mL straws (IMV, L'Aigle, France) in the cold gas, which was evaporated from liquid nitrogen, and thawed at 38°C for 10 s before examination.

### 2.3 Assessment of Sperm Motility and Quality

Motility analyses were performed using a computer‐assisted sperm analysis system with a Sperm Class Analyzer (SCA v.4.2) device (MICROPTIC S.L., Barcelona, Spain) using a Makler chamber (10‐μm depth; Sefi Medical Instruments, Haifa, Israel). Motility analysis was based on the examination of 25 consecutive digitized images obtained from three to five fields using a ×10 phase contrast objective. At least 500 spermatozoa were analyzed per sample using an image capture speed of 40 ms. Cell viability, plasma membrane integrity, and acrosome integrity were analyzed using a live/dead stain combination (SYBR‐14/propidium iodide [PI]; LIVE/DEAD Sperm Viability Kit; Molecular Probes Inc., Eugene, OR, USA), hypo‐osmotic swelling test (Ahmad et al. 2003), and fluorescein isothiocyanate‐labeled peanut agglutinin (FITC‐PNA; Vector Laboratories Inc., Burlingame, CA, USA), respectively, as described by Taniguchi et al. (2014). Mitochondrial integrity was assessed using fluorochrome 5,5′,6,6′‐tetrachloro‐1,1′,3,3′‐tetraethylbenzimidazolyl‐carbocyanine iodide (JC‐1; Molecular Probe Inc.), following the method described by Kaewma et al. (2021). The quality of frozen‐thawed spermatozoa was assessed before culture and after 5 h of culture.

### 2.4 Oocyte Collection and IVM

Oocyte collection and IVM were performed as described by Namula et al. (2020). Briefly, ovaries of prepubertal crossbred gilts (Landrace × Large White × Duroc breeds) were collected from a local slaughterhouse. Cumulus‐oocyte complexes (COCs) from follicles (3–6‐mm diameter) were collected using a surgical blade. Approximately 50 COCs were cultured in 500 μL of IVM medium comprising TCM‐199 with Earle's salts (Thermo Fisher Scientific, Waltham, MA, USA), supplemented with 10% (v/v) porcine follicular fluid, 0.6‐mM cysteine, 50‐μM sodium pyruvate, 2‐mg/mL d‐sorbitol (Fujifilm Wako), 50‐μM β‐mercaptoethanol (Fujifilm Wako), 10‐IU/mL equine chorionic gonadotropin (eCG, Kyoritsu Seiyaku Corporation, Tokyo, Japan), 10‐IU/mL human chorionic gonadotropin (hCG, Kyoritsu Seiyaku Corporation), 20‐μg/mL epidermal growth factor (EGF), and 50‐μg/mL gentamicin. The COCs were cultured at 39°C in a humidified incubator with 5% CO_2_ in IVM medium supplemented with hormones (eCG and hCG) and EGF for the first 22 h and then in IVM medium without hormones and EGF for an additional 22 h.

### 2.5 IVF and Culture

After IVM, the oocytes were subjected to IVF as described previously (Namula et al. 2020). Briefly, thawed spermatozoa were transferred to 6‐mL porcine fertilization medium (PFM; Research Institute for the Functional Peptides, Yamagata, Japan) and washed by centrifuging at 550 ×*g* for 5 min. The sperm pellet was resuspended in PFM to achieve a concentration of 2.0 × 10^6^ cells/mL. The spermatozoa (250 μL) were then added to 250‐μL PFM containing 50 mature oocytes in 4‐well dishes. The final sperm concentration was adjusted to 1 × 10^6^ cells/mL. The oocytes were then co‐incubated for 5 h at 39°C in a humidified incubator with 5% CO_2_, 5% O_2_, and 90% N_2_. After co‐incubation, the cumulus cells and the attached spermatozoa were detached from the inseminated zygotes by mechanical pipetting. Approximately 50 denuded zygotes were subsequently cultured in 500‐μL porcine zygote medium (PZM‐5; Research Institute for the Functional Peptides) overlaid with mineral oil in 4‐well dishes.

To assess oocyte fertilization, some zygotes were mounted on glass slides at 10 h after insemination and fixed with acetic acid:ethanol (1:3 v/v) for 48–72 h. The fixed zygotes were stained with acetic orcein (1% orcein in 45% acetic acid) and examined by phase contrast microscopy. Oocytes containing both female and male pronuclei were considered fertilized and were categorized as normal or polyspermic based on the number of swollen sperm heads and/or pronuclei in the cytoplasm (Do et al. 2015).

### 2.6 In Vitro Culture

The remaining zygotes were cultured continuously in vitro at 39°C in a humidified incubator with 5% CO_2_, 5% O_2_, and 90% N_2_. All cleaved embryos were transferred to 500‐μL porcine blastocyst medium (PBM; Research Institute for the Functional Peptides) after 72 h of insemination and cultured for an additional 4 days to evaluate their ability to develop to the blastocyst stage. To evaluate the total cell number in blastocysts, all embryos at the blastocyst and expanded blastocyst stages were fixed and stained with Hoechst 33342 at the end of the culture (Do et al. 2015).

### 2.7 Experimental Design

#### 2.7.1 Experiment 1

The effect of ZnCl_2_ supplementation during the in vitro culture of post‐thaw spermatozoa on their quality was examined using frozen‐thawed spermatozoa from boar A. The quality parameters of post‐thaw spermatozoa were assessed before culture and after 5 h of culture at 39°C in a humidified incubator with 5% CO_2_, 5% O_2_, and 90% N_2_ in PFM supplemented with 0‐ (control), 1‐, 10‐, and 20‐μg/mL ZnCl_2_ (Fujifilm Wako). Each experiment was repeated five times.

#### 2.7.2 Experiment 2

We then examined the effects of ZnCl_2_ supplementation during IVF on the fertilization and embryo development of porcine oocytes. In vitro‐matured oocytes were co‐incubated with frozen‐thawed spermatozoa from boar A in PFM supplemented with 0‐, 1‐, 10‐, or 20‐μg/mL ZnCl_2_. After IVF, the oocytes were cultured as described above. Each experiment was repeated seven times.

#### 2.7.3 Experiment 3

To verify the results of Experiment 2 with frozen‐thawed spermatozoa of varying quality, the effects of ZnCl_2_ on fertilization and development were tested using spermatozoa from three different boars (B–D). A ZnCl_2_ concentration of 1 μg/mL was determined to be the best for porcine oocyte fertilization and embryo development in Experiment 2. Therefore, in Experiment 3, in vitro‐matured oocytes were co‐incubated with frozen‐thawed spermatozoa in PFM supplemented with and without 1‐μg/mL ZnCl_2_. After IVF, the oocytes were cultured as described above. Each experiment was repeated five times.

#### 2.7.4 Experiment 4

We then examined the effects of ZnCl_2_ supplementation on the fertilization of porcine oocytes co‐incubated with different metal complexes of EDTA. In vitro‐matured oocytes were co‐incubated with frozen‐thawed spermatozoa from boar A for 5 h in PFM supplemented with 1‐μg/mL ZnCl_2_, with or without 410.3‐μg/mL calcium (Ca)‐EDTA (Dojindo, Kumamoto, Japan) or 471.64‐μg/mL zinc (Zn)‐EDTA (Dojindo). After IVF, oocyte fertilization was evaluated as described above. Each experiment was repeated four times.

### 2.8 Statistical Analysis

The examined parameters were subjected to analyses of variance (ANOVA) using the general linear model procedure in SAS for Windows, version 9.1 (SAS Institute, Cary, NC, USA). In Experiment 3, the statistical model included boar, ZnCl_2_ supplementation, and two‐way interactions. If the interactions were not significant, they were excluded from the model but retained to test the effects of boar or ZnCl_2_. Differences were considered statistically significant at *p* < 0.05.

## 3 Results

In Experiment 1, the effects of ZnCl_2_ concentration on the quality of frozen‐thawed spermatozoa after culturing for 5 h were evaluated. No significant differences were found in the percentages of motility, sperm viability, plasma membrane integrity, acrosome integrity, and mitochondrial integrity among spermatozoa cultured for 5 h, irrespective of ZnCl_2_ concentration (Table 1). Moreover, all parameters of spermatozoa cultured for 5 h were significantly decreased compared with those determined before culture (*p* < 0.05).

**TABLE 1:** Effects of zinc chloride (ZnCl_2_) concentration on the quality of frozen‐thawed spermatozoa after 5 h of incubation^a^.

Concentration of ZnCl_2_ (μg/mL)^b^	Motility (%)		Viability (%)	Plasma membrane integrity (%)	Acrosome integrity (%)	Mitochondrial integrity (%)
	Total	Progressive				
Before	44.7 ± 2.9, a	20.4 ± 1.4, a	23.5 ± 3.1, a	22.0 ± 1.6, a	92.1 ± 0.9, a	21.8 ± 1.9, a
Control	25.9 ± 1.9, b	7.9 ± 0.4, b	9.6 ± 1.5, b	10.7 ± 1.1, b	30.9 ± 4.1, b	7.4 ± 0.7, b
1	24.3 ± 2.4, b	7.1 ± 0.4, b	8.9 ± 0.8, b	11.1 ± 1.3, b	30.1 ± 5.1, b	8.2 ± 1.3, b
10	23.2 ± 2.0, b	6.8 ± 0.5, b	10.5 ± 0.9, b	11.3 ± 1.7, b	25.3 ± 3.7, b	7.1 ± 1.4, b
20	20.8 ± 2.0, b	6.8 ± 0.6, b	9.1 ± 1.2, b	11.7 ± 1.7, b	29.2 ± 2.6, b	8.7 ± 1.5, b

*Note:* Values with different letters (a, b) in the same column at each incubation time differ significantly (*p* < 0.05).

^a^ Five replicate trials were performed. Percentages are expressed as the mean ± SEM.

^b^ The quality parameters of post‐thaw spermatozoa from boar A were assessed before incubation (before) and after 5 h of incubation in the porcine fertilization medium supplemented with 0 (control), 1, 10, and 20 μg/mL of ZnCl_2_.

In Experiment 2, we examined the effects of ZnCl_2_ concentration during IVF on the fertilization and embryo development of porcine oocytes fertilized with frozen‐thawed spermatozoa. Despite no significant differences in the percentages of total fertilization, supplementation with 1‐μg/mL ZnCl_2_ significantly decreased the percentage of polyspermic fertilization compared with that in the control and 20‐μg/mL ZnCl_2_ supplementation groups (*p* < 0.05) (Table 2). Moreover, the percentage of blastocyst formation was significantly higher (*p* < 0.05) in the 1‐μg/mL ZnCl_2_ supplementation group than in the other supplementation groups. No significant differences in the percentages of cleaved embryos and total cell numbers in blastocysts were observed among the groups.

**TABLE 2:** Effects of zinc chloride (ZnCl_2_) concentration on the fertilization and development of porcine oocytes with cryopreserved spermatozoa^a^.

Concentration of ZnCl_2_ (μg/mL)^b^	No. of examined oocytes	No. (%) of oocytes^c^		No. of examined oocytes	No. (%) of embryos		Total cell number in blastocysts
		Total fertilization	Polyspermy		Cleaved	Developed to blastocysts	
0	100	82 (81.5 ± 4.8)	28 (33.3 ± 4.4), a	260	214 (80.5 ± 6.2)	30 (11.8 ± 1.6), a	55.2 ± 5.4
1	106	95 (89.2 ± 4.2)	14 (13.5 ± 2.8), b	278	223 (79.9 ± 1.7)	48 (17.9 ± 2.7), b	54.6 ± 4.2
10	110	95 (85.2 ± 2.2)	24 (22.1 ± 6.0), a,b	268	204 (76.0 ± 2.4)	20 (7.5 ± 1.9), a	55.5 ± 4.8
20	107	97 (90.5 ± 3.0)	32 (29.9 ± 5.8), a	287	206 (70.8 ± 4.4)	21 (7.2 ± 1.6), a	55.3 ± 5.4

*Note:* Values with different letters (a,b) in the same column differ significantly (*p* < 0.05).

^a^ Seven replicate trials were performed. Percentages are expressed as the mean ± SEM.

^b^ In vitro‐matured oocytes were co‐incubated with frozen‐thawed spermatozoa from boar A for 5 h in the porcine fertilization medium supplemented with 0 (control), 1, 10, and 20 μg/mL of ZnCl_2_.

^c^ The fertilization rate was defined as the ratio of the number of fertilized oocytes to the total number of examined oocytes. The polyspermic fertilization rate was defined as the ratio of the number of monospermic oocytes to the total number of fertilized oocytes.

In Experiment 3, the results of Experiment 2 were verified using different spermatozoa with varying quality. When the quality of the frozen‐thawed spermatozoa from boars B, C, and D was assessed before IVF use, the total motility percentages were 15.5%, 31.5%, and 59.6%, and the progressive motility percentages were 6.4%, 21.4%, and 42.1%, respectively. Further, sperm viability was 10.9%, 30.2%, and 30.8%; plasma membrane integrity was 8.4%, 6.7%, and 8.6%; and acrosome activity was 93.4%, 90.7%, and 92.3%, respectively. We then evaluated the effects of ZnCl_2_ supplementation during IVF on the fertilization and development of porcine oocytes fertilized with frozen‐thawed spermatozoa from three different boars. No significant boar × ZnCl_2_ supplementation interactions were observed for fertilization, embryo development, and total cell number. With each boar, although supplementation with 1‐μg/mL ZnCl_2_ did not affect the percentages of total fertilization, ZnCl_2_ supplementation significantly decreased the percentages of polyspermic fertilization compared with those in the control group (*p* < 0.05; Table 3). However, no significant differences were found in the percentages of embryo development and total cell number in blastocysts between the control and supplementation groups.

**TABLE 3:** Effects of zinc chloride (ZnCl_2_) supplementation on the fertilization and development of porcine oocytes with cryopreserved spermatozoa from different boars^a^.

Boar^b^	Group^c^	No. of examined oocytes	No. (%) of oocytes^d^		No. of examined oocytes	No. (%) of embryos		Total cell number in blastocysts
			Total fertilization	Polyspermy		Cleaved	Developed to blastocysts	
B	CT	41	38 (93.5 ± 4.2), a,b	22 (55.1 ± 8.3), a	235	193 (82.5 ± 1.7)	24 (10.6 ± 1.8), a	45.5 ± 3.5
	Zn	42	41 (98.0 ± 2.0), a	6 (13.5 ± 3.9), b	243	206 (84.8 ± 3.1)	28 (11.6 ± 0.6), a	45.9 ± 2.7
C	CT	47	39 (82.1 ± 6.3), b	22 (57.4 ± 7.8), a	236	197 (83.0 ± 5.3)	0 (0), b	—
	Zn	48	44 (92.0 ± 5.8), a,b	10 (22.9 ± 3.4), b	250	199 (79.4 ± 4.1)	4 (1.6 ± 1.0), b,c	40.0 ± 4.6
D	CT	45	41 (91.6 ± 4.1), a,b	22 (54.6 ± 8.6), a	246	208 (84.7 ± 2.4)	11 (4.5 ± 0.4), c,d	48.0 ± 5.4
	Zn	44	43 (98.0 ± 2.0), a	12 (29.5 ± 7.8), b	232	189 (81.1 ± 4.8)	15 (6.4 ± 2.6), d	42.8 ± 4.8

*Note:* Values with different letters (a–d) in the same column differ significantly (*p* < 0.05).

^a^ Five replicate trials were performed. Percentages are expressed as the mean ± SEM.

^b^ Frozen‐thawed spermatozoa from three different boars were used for in vitro fertilization.

^c^ In vitro‐matured oocytes were co‐incubated with frozen‐thawed spermatozoa for 5 h in porcine fertilization medium supplemented with 1‐μg/mL ZnCl_2_ (Zn) or without it (CT).

^d^ The fertilization rate was defined as the ratio of the number of fertilized oocytes to the total number of examined oocytes. The monospermic fertilization rate was defined as the ratio of the number of monospermic oocytes to the total number of fertilized oocytes.

**FIGURE 1:**
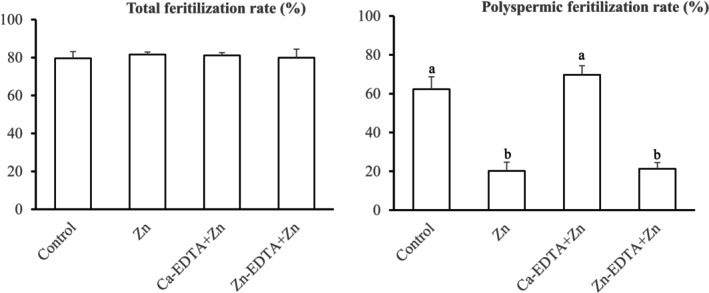
Effects of zinc chloride (ZnCl_2_) supplementation on the fertilization of porcine oocytes co‐incubated with different complexes of the metal chelator, ethylenediaminetetraacetic acid (EDTA). In vitro‐matured oocytes were co‐incubated with frozen‐thawed spermatozoa for 5 h in porcine fertilization medium supplemented with 1‐μg/mL ZnCl_2_ (Zn) or in combination with 410.3‐μg/mL Ca‐EDTA (Ca‐EDTA + Zn) or 471.64‐μg/mL Zn‐EDTA (Zn‐EDTA + Zn). As a control, oocytes were co‐incubated with frozen‐thawed spermatozoa without Zn and EDTA. Four replicate trials were performed. Data are expressed as the mean ± SEM. The total fertilization rate was defined as the ratio of the number of fertilized oocytes to the total number of examined oocytes (number of examined oocytes in each group, 118–136). The polyspermic fertilization rate was defined as the ratio of the number of monospermic oocytes to the total number of fertilized oocytes (number of fertilized oocytes in each group, 93–108). ^a–b^Bars with different superscript letters differ significantly (*p* < 0.05).

In Experiment 4, we investigated the effects of ZnCl_2_ supplementation on fertilization during co‐incubation with the metal chelator EDTA (Figure 1). EDTA addition did not affect the percentages of total fertilization (79.6%–81.6%). However, when compared with those in the control group (62.3%), co‐incubation with Zn‐EDTA (21.3%), but not Ca‐EDTA (69.7%), significantly reduced the percentages of polyspermic fertilization of oocytes similar to those with ZnCl_2_ alone (20.2%) (*p* < 0.05).

## 4 Discussion

The success of porcine IVF depends on generating normal embryos. The high incidence of chromosomal abnormalities, which result from polyspermy or the fertilization of oocytes that have halted at the diploid stage, seriously compromises the normalcy of in vitro fertilized embryos (Dang‐Nguyen et al. 2011; Himaki et al. 2025). In this study, we found that ZnCl_2_ supplementation during IVF decreased polyspermic fertilization without reducing the total fertilization rate.

The main parameters for evaluating sperm quality include sperm motility, sperm morphology, and sperm quality, defined by biomarkers such as sperm viability, cell membrane integrity, acrosome structure integrity, and mitochondrial integrity (Chen et al. 2021). Moreover, previous studies on porcine IVF have demonstrated that sperm penetration as well as polyspermic fertilization occurs during the first 2 h after insemination, with the highest rate of monospermic fertilization observed approximately 6‐h postinsemination (Marchal et al. 2002). These results indicate that although fertilization begins early, a significant proportion of oocytes require approximately 6 h to complete fertilization, and the mechanisms preventing polyspermic fertilization remain active for several hours after fertilization. Therefore, herein, sperm parameters were examined at 5‐h postculture to evaluate sperm function reflecting the in vitro environment and timing. We found that the concentration of ZnCl_2_ had no significant effects on the sperm parameters when evaluated after 5 h of culture, the same time as IVF. However, when the oocytes and spermatozoa were fertilized in vitro in a medium supplemented with 1‐μg/mL ZnCl_2_, the percentages of polyspermic fertilization decreased without reducing the total fertilization rate. These results indicate that ZnCl_2_ supplementation during IVF improves monospermic fertilization. To date, the rate of polyspermy has been reported to be affected by the media used (Coy and Romar 2002). Zinc plays an important role in capacitation and fertilization by inducing a sperm zinc ion efflux, where the role of zinc shifts to inhibiting proteinases to prevent polyspermy (Kerns et al. 2020). Additionally, the zinc spark, occurring simultaneously or possibly preceding the oocyte cortical reaction (the primary anti‐polyspermy defense mechanism), is suggested to function as a rapid polyspermy block (Que et al. 2017). The release of cortical granules is an important step in the ZP block to prevent polyspermy (Wang et al. 1998). Zinc is suggested to affect the ZP block by activating the release of cortical granules (Que et al. 2017). The ZP block mechanism occurs after sperm‐oocyte binding, and a large amount of zinc is released in the region surrounding the oocyte, but this zinc efflux event activates hardening of the ZP glycoprotein matrix to prevent sperm from binding to the fertilized oocyte (Que et al. 2017). Therefore, zinc supplementation during IVF may improve the ZP‐blocking mechanism of oocytes for monospermic fertilization rather than enhancing sperm fertilization capacity.

In the present study, when examining the effects of zinc supplementation using spermatozoa from different boars, ZnCl_2_ supplementation (1 μg/mL) reduced the percentage of polyspermic fertilization, regardless of the boar sperm tested, but did not improve embryo development and the quality of blastocysts. In cattle, polyploid embryos are reported to be eliminated before the 8‐cell stage when the main embryonic genome is activated, but the proportion of mixed polyploid embryos produced in vitro increases during culture until the blastocyst stage (Viuff et al. 1999). Similarly, pig polyspermic embryos have been known to develop to the blastocyst stage at the same percentage as normal embryos (Han et al. 1999). Therefore, determining the incidence of embryos that develop to the blastocyst stage may not be a suitable method for assessing the rate of normal fertilization of porcine embryos (Funahashi 2003). In the present study, the improvement in embryo development achieved by zinc supplementation using spermatozoa from three different boars was not as significant as that observed when using spermatozoa from boar A. A potential explanation for this difference is that the blastocyst formation rate was determined by including embryos resulting from polyspermic fertilization, which may have masked any significant effects. Moreover, in porcine IVF, notable variations in fertilization and embryo development rates have been observed among sperm‐donor boars (Gil et al. 2008). These variations are posited to result from the characteristics of sperms and the conditions of co‐culture between sperms and oocytes, indicating that not all boars exhibit uniform responses to IVF conditions (Gil et al. 2008). It is plausible that sperms from specific boars exhibit distinct responses to Zn^2+^ supplementation, which may account for the enhanced blastocyst formation rates observed in spermatozoa from boar A following ZnCl_2_ supplementation. On the other hand, ZnCl_2_ leached from glassware utilized in whole embryo culture has been reported to reach concentrations ≥ 6 μM, which, even at such low levels, has been demonstrated to impact mouse embryo development negatively (Yao et al. 2025). Moreover, it has been suggested that sensitivity to ZnCl_2_ varies among different animal species (Yao et al. 2025). In the present study, the effects of elevated ZnCl_2_ concentrations (approximately 7–147 μM corresponding to 1–20 μg/mL) during IVF were investigated; however, no significant reduction in pig embryo development was detected compared to that in the control group. These results indicate that the toxicity of ZnCl_2_ might have no apparent effects because of differences in species and restricted exposure times. Therefore, our results indicate that zinc supplementation during IVF may not influence either embryo development or embryo quality.

Ions that form higher stability complexes with EDTA are more likely to be chelated than those that form lower stability complexes (Hirata 1981; Flora and Pachauri 2010). With Ca‐EDTA addition, other trace element ions (Zn^2+^, Cu^2+^, and Fe^3+^) are chelated, reducing the concentrations of these ions. In contrast, Zn‐EDTA addition cannot inhibit the action of Ca^2+^ and Zn^2+^ but reduces other higher stability ions (Cu^2+^ and Fe^3+^). In the present study, we found that Ca‐EDTA addition did not reduce polyspermic fertilization despite the addition of ZnCl_2_; however, Zn‐EDTA addition decreased the percentage of polyspermic fertilization similar to the addition of ZnCl_2_ alone. Moreover, the addition of EDTA did not affect the percentages of total fertilization. On the other hand, the same boar was used in Experiments 2 and 4, but polyspermic fertilization rates in the control group differed (33.3% vs. 62.3%). This difference may be due to variations in IVF conditions, including sperm preparation, oocyte quality, and environmental factors (Poniedziałek‐Kempny 2020). However, ZnCl_2_ supplementation (1 μg/mL) consistently suppressed polyspermic fertilization in both experiments. In Experiment 2, the polyspermy rate decreased from 33.3% (0 μg/mL) to 13.5% (1 μg/mL), and in Experiment 4, from 62.3% (control) to 20.2% (Zn), reducing polyspermic fertilization to approximately one‐third of control values. Regardless of control value variations, this consistency across experiments demonstrates the reliability of the anti‐polyspermy effect of ZnCl_2_ during IVF. Therefore, our results indicate that the decrease in polyspermic fertilization is attributed to the presence of ZnCl_2_ during IVF, marking an increase in monospermic fertilization.

In conclusion, this study demonstrates that supplementation with 1‐μg/mL ZnCl_2_ during porcine IVF decreases polyspermic fertilization but does not affect embryo development. Moreover, the improvement in fertilization with ZnCl_2_ supplementation may be because of an improvement in oocyte competence related to ZP‐blocking mechanisms rather than an improvement in sperm quality as for the pig. To further elucidate this hypothesis, future studies should focus on clarifying how zinc modulates biochemical and structural changes in the ZP that contribute to polyspermy prevention, including potential effects on cortical granule exocytosis and ZP hardening.

## Conflicts of Interest

The authors declare no conflicts of interest.
